# Reinstatement of *Lappula betpakdalensis* (Boraginaceae) based on phylogenomic and morphological evidence

**DOI:** 10.3897/phytokeys.279.207568

**Published:** 2026-09-09

**Authors:** Dan-Hui Liu

**Affiliations:** 1 State Key Laboratory of Ecological Safety and Sustainable Development in Arid Lands, Xinjiang Institute of Ecology and Geography, Chinese Academy of Sciences, Urumqi 830011, China tate Key Laboratory of Ecological Safety and Sustainable Development in Arid Lands, Xinjiang Institute of Ecology and Geography, Chinese Academy of Sciences Urumqi China; 2 China-Tajikistan Belt and Road Joint Laboratory on Biodiversity Conservation and Sustainable Use, Xinjiang Institute of Ecology and Geography, Chinese Academy of Sciences, Urumqi 830011, China China-Tajikistan Belt and Road Joint Laboratory on Biodiversity Conservation and Sustainable Use, Xinjiang Institute of Ecology and Geography, Chinese Academy of Sciences Urumqi China

**Keywords:** Angiosperms353, chloroplast genomes, integrative taxonomy, *
Lappula
*, taxonomic revision

## Abstract

*Lappula lasiocarpa* is highly distinctive and easily distinguished from other congeners within *Lappula* owing to its saccate nutlet wings with entire margins densely covered in pubescence. Following the taxonomic revision of *Lappula*, the Flora of China treated *L. betpakdalensis* as a synonym of *L. lasiocarpa*. However, a comparison of the type specimens of these two taxa reveals clear morphological differences. Although both species produce distinctly winged nutlets, the wings of *L. betpakdalensis* are dentate, whereas those of *L. lasiocarpa* are entire and densely pubescent along their margins. These striking differences in nutlet morphology cast doubt on their conspecific status. In the present study, phylogenomic analyses based on chloroplast genomes and Angiosperms353 datasets placed both *L. betpakdalensis* and *L. lasiocarpa* within the same major clade (Clade Fa in the chloroplast phylogeny and Clade VIIb in the nuclear phylogeny), together with their close relatives *L. macrantha*, *L. semiglabra*, and *L. pavlovii*. Nevertheless, within this clade, *L. betpakdalensis* forms a strongly supported independent lineage distinct from *L. lasiocarpa* (LPP = 1.00, BS = 100). Furthermore, morphological comparisons confirmed that, in addition to the contrasting nutlet wing margins (entire vs. dentate), corolla size is another key diagnostic character distinguishing the two species. Specifically, *L. betpakdalensis* has a corolla 3–4 mm in diameter, whereas that of *L. lasiocarpa* measures 5–6 mm. Taken together, the phylogenomic and morphological evidence does not support the synonymization of *L. betpakdalensis* under *L. lasiocarpa*. Accordingly, *L. betpakdalensis* is reinstated as a distinct species.

## Introduction

The genus *Lappula* Moench belongs to the subfamily Cynoglossoideae and the tribe Rochelieae (Chacón et al. 2016). It comprises approximately 50–80 species distributed across Eurasia, North Africa, the Americas, and Australia, with its center of diversity in Central Asia (Wang 1981; Ovczinnikova 2005, 2021; Weigend et al. 2016). The genus is characterized by a subulate gynobase and nutlets, which bear either marginal glochids or wings (Popov 1953a; Riedl 1967; Zhu et al. 1995; Weigend et al. 2016). Although the circumscription of *Lappula* was widely accepted for a long time, recent molecular phylogenetic studies have demonstrated that the genus, as traditionally circumscribed, is polyphyletic (Nazaire and Hufford 2012; Huang et al. 2013; Khoshsokhan-Mozaffar et al. 2018). Consequently, in accordance with the principle of monophyly, the taxonomic relationships among *Lappula* and the allied genera *Rochelia*, *Pseudolappula*, and *Lepechiniella* have been reassessed and revised (Khoshsokhan-Mozaffar et al. 2018; Liu et al. 2021, 2025a).

*Lepechiniella* is the genus most closely related to *Lappula*. It was established by Popov (1953a), who considered *Lepechiniella* to differ from *Lappula* only in having a shorter gynobase and winged nutlets. However, Nabiev (1986) did not accept this view, arguing that *Lepechiniella* does not represent a distinct genus but should instead be included within *Lappula*. Accordingly, he transferred all species of *Lepechiniella* to *Lappula*. Because the name *Lappula balchaschensis* Popov ex Golosk. had already been validly published (Goloskokov 1975), transferring *Lepechiniella balchaschensis* Popov to *Lappula* would have created a later homonym. To avoid this, Nabiev proposed the replacement name *L. betpakdalensis* Nabiev. Subsequently, some authors adopted Nabiev’s broader circumscription of *Lappula* (Zhu et al. 1995), whereas others continued to recognize *Lepechiniella* as a distinct genus (Riedl 1967; Ovczinnikova 2005; Weigend et al. 2016). More recently, multiple independent molecular phylogenetic studies have supported Nabiev’s taxonomic treatment, demonstrating that several species formerly assigned to *Lepechiniella*, including the type species, are nested within *Lappula* (Khoshsokhan-Mozaffar et al. 2018; Liu et al. 2025a).

In their revision of *Lappula* in China, Zhu et al. (1995) accepted Nabiev’s generic circumscription and transferred the only Chinese species originally described in *Lepechiniella*, *L. lasiocarpa* W.T. Wang, to *Lappula*. They also treated *L. betpakdalensis* Nabiev, *L. balchaschensis* Popov, and *L. omphaloides* (Schrenk) Popov var. *balchaschensis* Popov as synonyms of *L. lasiocarpa*. *Lappula lasiocarpa* is morphologically distinctive within the genus in having nutlets in which the wing on the margin of the dorsal disc curves upward to form a saccate structure; the wing is entire, with a densely pubescent margin. These unique nutlet characteristics allow the species to be readily identified, even in the absence of other diagnostic features.

Examination of the type specimens of *L. lasiocarpa* and *L. betpakdalensis* revealed clear differences in their nutlet morphology. The nutlets of *L. lasiocarpa* have entire margins, with the wing constricted distally to form a saccate structure, whereas those of *L. betpakdalensis* also bear distinct wings, which are dentate, with the teeth usually terminating in glochids. Nutlet morphology is a key character for infrageneric classification and species delimitation in *Lappula* (Popov 1953a; Riedl 1967; Zhu et al. 1995; Ovczinnikova 2021; Liu et al. 2021). Although nutlet morphology is highly variable within the genus, the variation observed in *L. lasiocarpa* is limited to broad-winged and narrow-winged forms (Liu et al. 2025b). In contrast, the nutlet morphology of *L. betpakdalensis* falls outside the range of variation observed in *L. lasiocarpa*. Accordingly, *L. betpakdalensis* and *L. lasiocarpa* are hypothesized to represent distinct taxonomic entities.

To test this hypothesis, samples of *L. betpakdalensis* were obtained from the Moscow University Herbarium (MW) and the Vascular Plants Herbarium of the Komarov Botanical Institute, Russian Academy of Sciences (LE), and incorporated into the phylogenomic framework of *Lappula* established by Liu et al. (2025a). Using data generated by deep genome skimming, complete chloroplast genomes were assembled, and Angiosperms353 nuclear loci were recovered to clarify the phylogenetic relationship between *L. lasiocarpa* and *L. betpakdalensis*. In addition, specimens collected during field surveys and from herbarium collections were examined, and detailed morphological comparisons were conducted between the two species. By integrating phylogenomic and morphological evidence, the taxonomic status of *L. lasiocarpa* and *L. betpakdalensis* was evaluated.

## Materials and methods

### Taxon sampling, DNA extraction, and sequencing

Raw sequencing data for 64 *Lappula* samples were downloaded from the dataset of Liu et al. (2025a). In addition, four samples of *L. betpakdalensis* were included in this study, including two obtained from the Moscow University Herbarium (MW) and two from the Vascular Plants Herbarium of the Komarov Botanical Institute, Russian Academy of Sciences (LE). These samples were sent to Novogene Biotechnology (Tianjin, China) for DNA extraction, library construction, and high-throughput sequencing, yielding approximately 10 Gb of raw sequencing data per sample. Raw sequencing reads were filtered using Trimmomatic v. 0.39 (Bolger et al. 2014) with the same parameters as those described by Liu et al. (2025a). A complete list of the sampled taxa and their voucher information is provided in Suppl. material 1: table S1.

### Assembly of chloroplast genomes and Angiosperms353 genes

Chloroplast genomes were assembled from the Trimmomatic-filtered reads using GetOrganelle v. 1.7.5 (Jin et al. 2020) with default parameters. The assembled plastomes were annotated using PlastidHub (Zhang et al. 2025). For subsequent phylogenetic analyses, two datasets were generated: (1) complete chloroplast genomes with one inverted repeat (IR) region removed and (2) the corresponding coding sequences (CDS) extracted from these plastomes. The Angiosperms353 nuclear loci were recovered using the mega353 target file (McLay et al. 2021) and the HybPiper v. 2.3.3 pipeline (Johnson et al. 2016). Assembly performance was evaluated using the HybPiper utilities: hybpiper stats to calculate gene recovery rates, hybpiper recovery_heatmap to visualize gene recovery patterns, and hybpiper retrieve_sequences to extract successfully assembled sequences for downstream phylogenomic analyses.

### Phylogenomic analyses

Because this study focused on the phylogenetic relationship between *L. lasiocarpa* and *L. betpakdalensis*, no outgroups outside *Lappula* were included. Instead, following Khoshsokhan-Mozaffar et al. (2018) and Liu et al. (2025a), species of *Lappula* sect. *Sclerocaryum* A.DC. (*L. spinocarpos* (Forssk.) Asch. ex Kuntze, *L. balchaschensis*, *L. deserticola* C.J.Wang, and *L. krylovii* Ovczinnikova, Pjak & A.L.Ebel) were designated as the outgroup for all phylogenomic analyses.

For the chloroplast dataset, both complete plastome sequences (with one inverted repeat region removed) and CDS datasets were analyzed. Sequences were aligned using MAFFT v. 7.505 (Katoh and Standley 2013) and trimmed with TrimAl v. 1.2 (Capella-Gutiérrez et al. 2009) using default parameters. The CDS alignments were concatenated in PhyloSuite (Zhang et al. 2020). The best-fitting nucleotide substitution model was selected using ModelFinder v. 2.2.0 (Kalyaanamoorthy et al. 2017). Maximum likelihood (ML) analyses were conducted in IQ-TREE2 v. 2.2.0 (Minh et al. 2020), with branch support assessed using 1,000 SH-aLRT replicates and 1,000 ultrafast bootstrap replicates. Bayesian inference (BI) analyses were performed in MrBayes v. 3.2.7a (Ronquist et al. 2012) using two independent Markov chain Monte Carlo (MCMC) runs of 50,000,000 generations, with trees sampled every 5,000 generations. The first 25% of sampled trees were discarded as burn-in.

For the Angiosperms353 dataset, both concatenation- and coalescent-based approaches were employed. Each locus was aligned with MAFFT v. 7.505 and trimmed using TrimAl v. 1.2 under default settings. Genes with an average alignment length of less than 300 bp or taxon occupancy below 80% (i.e., represented by fewer than 55 samples) were excluded to generate a high-quality dataset for downstream analyses. For the concatenation approach, the retained loci were concatenated in PhyloSuite to produce a supermatrix. PartitionFinder2 (Lanfear et al. 2017) was used with the rcluster algorithm and the corrected Akaike information criterion (AICc) (Lanfear et al. 2014) to determine the optimal partitioning scheme and substitution models. The partitioned ML analysis was conducted in IQ-TREE2 v. 2.2.0, with branch support evaluated using 1,000 SH-aLRT replicates and 1,000 ultrafast bootstrap replicates. For the coalescent-based analysis, ML trees for each filtered locus were reconstructed in IQ-TREE2 v. 2.2.0 using 1,000 ultrafast bootstrap replicates. Branches with bootstrap support below 10% were collapsed using Newick Utilities before species tree inference. The resulting gene trees were analyzed with ASTRAL v. 5.7.8 (Zhang et al. 2018) to infer the species tree, with branch support estimated as local posterior probability (LPP). All phylogenetic trees were visualized and edited using iTOL (Letunic and Bork 2024).

### Morphological observation and comparison

A comprehensive morphological comparison of *L. lasiocarpa* and *L. betpakdalensis* was conducted based on specimens collected during field surveys in Xinjiang, China, together with physical specimens and digitized herbarium images from the Vascular Plants Herbarium of the Komarov Botanical Institute, Russian Academy of Sciences (LE), the Moscow University Herbarium (MW), the Central Siberian Botanical Garden, Siberian Branch of the Russian Academy of Sciences (NSK), the Institute of Botany, Chinese Academy of Sciences (PE), and the Xinjiang Institute of Ecology and Geography, Chinese Academy of Sciences (XJBI). The two species were compared using a comprehensive set of morphological characters, including life form, indumentum, corolla morphology, and diagnostic nutlet characters.

## Results

### Assembly of chloroplast genomes and Angiosperms353 genes

Using deep genome skimming data from 64 *Lappula* samples reported by Liu et al. (2025a), together with four newly sequenced samples, chloroplast genomes were successfully assembled for all 68 samples using GetOrganelle. The assembled plastomes ranged from 145,677 bp in *L. patula* (Lehm.) Menyh. to 147,837 bp in *L. deserticola*, with an average length of 147,147 bp. All plastomes exhibited the typical quadripartite structure, comprising a large single-copy (LSC) region (79,259–80,650 bp), a small single-copy (SSC) region (16,939–17,195 bp), and a pair of IR regions (24,639–25,015 bp each). The plastomes contained 128–130 genes, including 83–85 protein-coding genes, eight ribosomal RNA (rRNA) genes, and 37 transfer RNA (tRNA) genes. Detailed assembly statistics for each sample are provided in Suppl. material 1: table S2. For phylogenetic analyses, one copy of the IR region was removed from each plastome. The resulting complete plastome alignment comprised 68 samples and was 122,662 bp in length, whereas the concatenated CDS alignment was 65,549 bp long.

Using the HybPiper pipeline, Angiosperms353 loci were successfully recovered from all 68 samples. The number of recovered loci per sample ranged from 234 to 347, with an average of 324 loci. With the exception of a small number of loci that showed relatively low recovery rates (loci 5347, 5513, 5642, and 11 additional loci; Suppl. material 1: table S3), most loci were recovered with high efficiency. Detailed recovery statistics are provided in Suppl. material 2: fig. S1 and Suppl. material 1: tables S3, S4. The recovered loci ranged from 32 to 4,287 bp in length, with a mean length of 647 bp. After quality filtering, 274 high-quality loci were retained for subsequent phylogenomic analyses.

### Phylogenomic analyses

**Figure 1.**
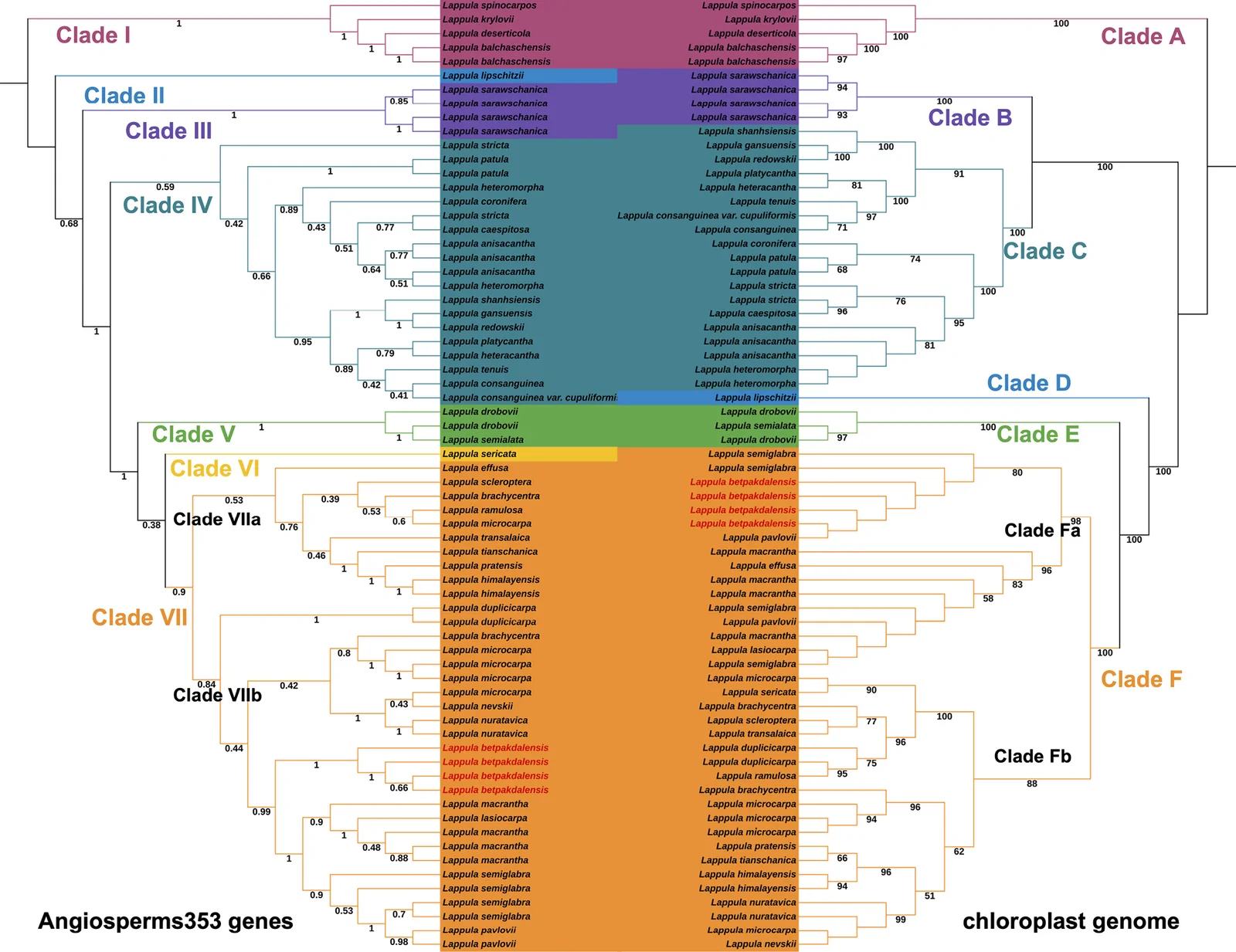
Coalescent-based species tree of *Lappula* inferred using ASTRAL-III from the Angiosperms353 dataset (left) and ML tree inferred from the chloroplast CDS dataset using IQ-TREE2 (right). LPP and BS values are shown below the branches. Major clades are distinguished by different colors. *L. betpakdalensis*, the focal species of this study, is highlighted in red. Its phylogenetic position is indicated in Clade VIIb in the nuclear phylogeny (left) and Clade Fa in the chloroplast phylogeny (right).

ML and BI phylogenetic trees were reconstructed using both complete plastome and CDS datasets. The four resulting trees showed largely congruent topologies. In the Results and Discussion, the ML tree inferred from the CDS dataset (Fig. 1) is presented, whereas the remaining trees are provided in Suppl. material 2: figs S3–S5.

The chloroplast phylogenies recovered six well-supported major clades. *Lappula spinocarpos*, *L. balchaschensis*, *L. deserticola*, and *L. krylovii* formed a clade sister to the remaining *Lappula* species. The two focal species, *L. lasiocarpa* and *L. betpakdalensis*, were both placed in Clade Fa, together with *L. macrantha* (Ledeb.) Gürke, *L. semiglabra* (Ledeb.) Gürke, *L. pavlovii* Golosk., and *L. effusa* D.H.Liu & W.J.Li, forming a strongly supported monophyletic lineage (bootstrap support [BS] = 100, posterior probability [PP] = 1.00). However, most internal nodes within Clade Fa received bootstrap support values below 50%, resulting in poor phylogenetic resolution among the species in this clade (Fig. 1). Consequently, although the chloroplast data clearly place *L. betpakdalensis* within Clade Fa, the phylogenetic relationship between *L. betpakdalensis* and *L. lasiocarpa* remains unresolved.

Phylogenetic trees inferred from the Angiosperms353 dataset using both concatenation- and coalescent-based approaches showed largely congruent topologies (Fig. 1, Suppl. material 2: fig. S2). Because the two analyses produced similar results, only the coalescent-based tree is described below. Seven major clades were recovered within *Lappula*. Clade I, comprising *L. spinocarpos*, *L. balchaschensis*, *L. deserticola*, and *L. krylovii*, was sister to the remaining *Lappula* species. *L. lasiocarpa* and *L. betpakdalensis* were placed within a strongly supported subclade of Clade VIIb (LPP = 1.00, BS = 100). The composition of this subclade was largely consistent with that of Clade Fa recovered in the chloroplast phylogeny. Compared with the chloroplast phylogeny, the Angiosperms353 phylogeny provided substantially higher support for the internal nodes within this subclade (LPP = 0.90–1.00, BS = 99–100), resulting in improved resolution of relationships among the constituent species. Within this subclade, the four samples of *L. betpakdalensis* formed a strongly supported monophyletic lineage (LPP = 1.00, BS = 100) that was sister to a clade comprising *L. lasiocarpa*, *L. macrantha*, *L. semiglabra*, and *L. pavlovii*. Within the latter clade, *L. lasiocarpa* and *L. macrantha* formed a well-supported clade (LPP = 0.90, BS = 99), which was sister to the clade consisting of *L. semiglabra* and *L. pavlovii* (LPP = 0.90, BS = 99).

### Morphological comparisons

**Figure 2.**
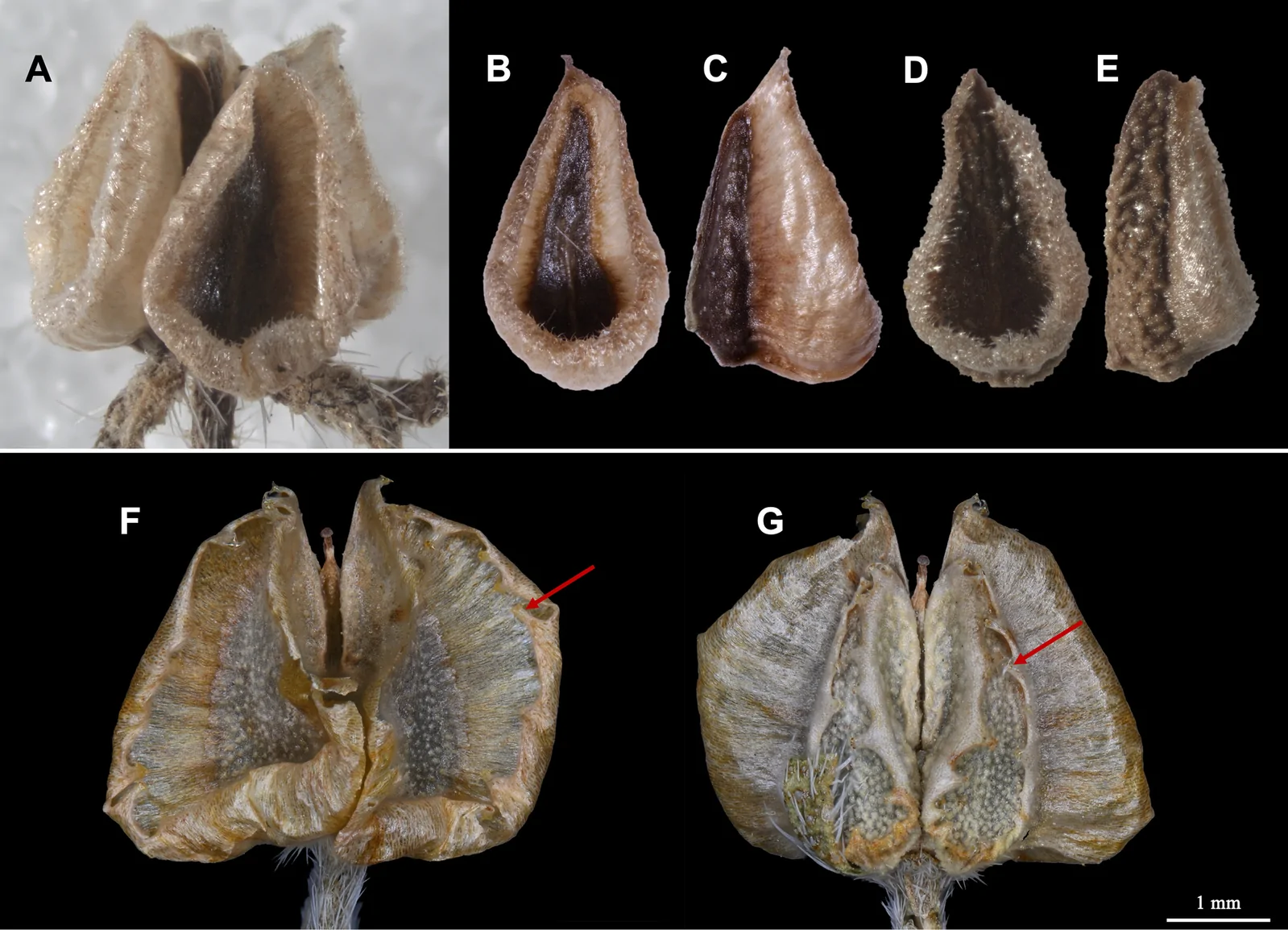
Fruit and nutlet morphology of *Lappula lasiocarpa* and *L. betpakdalensis*. **A–E**. *L. lasiocarpa*: **A**. Whole fruit; **B**. Abaxial view of a broadly winged nutlet; **C**. Lateral view of a broadly winged nutlet; **D**. Abaxial view of a narrowly winged nutlet; **E**. Lateral view of a narrowly winged nutlet. **F, G**. *L. betpakdalensis*: **F**. Fruit with broadly winged nutlets; **G**. Fruit with narrowly winged nutlets. Red arrows indicate the glochids along the margins of the nutlet wings.

A detailed morphological comparison was conducted between *L. lasiocarpa* and *L. betpakdalensis*. The two species share several morphological characters: both are annual herbs with spreading indumentum; their calyx lobes are narrowly ovate and shorter than the nutlets at fruiting; both produce heteromorphic nutlets; and the style does not exceed the nutlets in length. They differ primarily in corolla size and the morphology of the nutlet wing margins. *L. lasiocarpa* has a corolla approximately 5–6 mm in diameter and produces two types of heteromorphic nutlets (broadly winged and narrowly winged), both with entire, pubescent wing margins (Fig. 2A–E). By contrast, *L. betpakdalensis* has a smaller corolla (3–4 mm in diameter). It likewise produces broadly and narrowly winged nutlets, but their wing margins are dentate, with a glochid at the apex of each tooth (Fig. 2F–G). A detailed morphological comparison between *L. lasiocarpa* and *L. betpakdalensis* is provided in Table 1.

**Table 1.** Morphological comparisons of *Lappula betpakdalensis*, *L. lasiocarpa*, and *L. omphaloides*.

**Characters**		***L. betpakdalensis***	***L. lasiocarpa***	***L. omphalodes***
Life form		Annual	Annual	Annual or biennial
Stem	Height	10–30 cm	6–20 cm	7–20 cm
	Imdumentum	Spreading hair	Spreading hair	Appressed sericeous hair
Leaves	Basal leaves	Narrowly obovate, usually withering early	Narrowly obovate, usually withering early	Spatulate-linear or lanceolate, withering at fruiting
	Stem leaves	Linear; hirsute on both adaxial and abaxial surfaces	Linear; hirsute on both adaxial and abaxial surfaces	Spatulate-linear, hirsute on both adaxial and abaxial surfaces
Inflorescences		3–10 cm, bracts linear	ca. 8 cm, bracts linear	5–8 cm, bracts very small, inconspicuous
Flower	Pedicels	ca. 1 mm, elongating to 3 mm in fruit.	ca. 1 mm, elongating to 5 mm in fruit.	2–3 mm, elongating to 5–8 mm in fruit.
	Calyx	Lobes narrowly ovate, shorter than the nutlets at fruiting	Lobes narrowly ovate, shorter than the nutlets at fruiting	Lobes broadly linear, obtuse, shorter than the nutlets at fruiting
	Corolla	Blue, 3–4 mm	Blue, 5–6 mm	Blue, 5–6 (7) mm
Style		Not surpassing nutlets	Not surpassing nutlets	Exceeding nutlets
Nutlet		Heteromorphic (broadly and narrowly winged)	Heteromorphic (broadly and narrowly winged)	Homomorphic (broadly winged)
	Wings	Entire, pubescent along the margin	Dentate, with glochids at the tips	Dentate, with glochids at the tips

## Discussion

### Confirmed the systematic position of *L. betpakdalensis*

Phylogenomic analyses based on chloroplast genomes and Angiosperms353 loci clarify the phylogenetic placement of *L. betpakdalensis* for the first time. The species is closely related to *L. lasiocarpa*, *L. macrantha*, *L. semiglabra*, and *L. pavlovii* and should be placed in *Lappula* sect. *Macranthae* (Riedl) Ovczinnikova, as circumscribed by Ovczinnikova (2005). The results do not support the placement of *L. betpakdalensis* in *Lepechiniella* sect. Lophoptereae M.Pop. (Popov 1953a) or its assignment to *Lepechiniella* sect. *Kazachstanicae* Ovczinnikova (Ovczinnikova 2005).

Popov (1953a) divided *Lepechiniella* into two sections based on nutlet morphology: sect. *Holoptereae* M.Pop., characterized by entire-winged nutlets, and sect. Lophoptereae M.Pop., characterized by coarsely dentate-winged nutlets. He placed *L. betpakdalensis* in sect. Lophoptereae, consistent with the observation that its nutlets possess dentate wings, in contrast to the entire-winged nutlets of *L. lasiocarpa*. Building on Popov’s classification, Ovczinnikova (2005) established sect. *Kazachstanicae*, including *Lepechiniella balchaschensis*, *L. michaelis* Golosk., *L. omphaloides* (Schrenk) Popov, and *L. saurica* (Bajtenov & Kudab.) Ovczinnikova. However, the phylogenomic results do not support the recognition of *Lepechiniella* as a distinct genus. Instead, they support its inclusion within *Lappula*, consistent with the conclusions of Khoshsokhan-Mozaffar et al. (2018) and Liu et al. (2025a).

The phylogenetic placement of *L. betpakdalensis* within Clade Fa of the chloroplast phylogeny and Clade VIIb of the Angiosperms353 phylogeny (Fig. 1, Suppl. material 2: figs S2–S5) further supports its assignment to sect. *Macranthae*. Although Clade Fa additionally includes *L. effusa*, the remaining species are shared between the two phylogenies and correspond closely to the circumscription of sect. *Macranthae*. Liu et al. (2025b) also recognized this lineage as possessing a distinctive nutlet type, characterized by nutlets 2.5–3.5 mm long with a thickness-to-length (*T/L*) ratio of 0.10–0.20, resulting in dorsiventrally compressed nutlets. The measurements show that the nutlets of *L. betpakdalensis* are 2.2–2.7 mm long, with a *T/L* ratio of approximately 0.15, likewise exhibiting dorsiventral compression.

Taken together, evidence from chloroplast and nuclear phylogenomics, as well as comparative morphology of the nutlets, consistently supports the placement of *L. betpakdalensis* in *Lappula* sect. *Macranthae*.

### *L. betpakdalensis* and *L. lasiocarpa* represent two distinct taxonomic entities

*Lepechiniella balchaschensis* (currently accepted as *L. betpakdalensis*) was originally described as a variety of *L. omphaloides*. Popov (1953a) distinguished this taxon from *L. omphaloides* by its spreading indumentum, shorter and broader leaves, shorter pedicels and peduncles, a smaller corolla (approximately 3–4 mm in diameter), and narrower nutlet wings. He suggested that these morphological differences could warrant recognition at the species level and subsequently elevated it to species rank (Popov 1953b). Morphological comparison between *L. betpakdalensis* and *L. omphaloides* reveals that the two taxa represent distinct entities; in addition to the diagnostic morphological traits outlined above, the stigma of *L. betpakdalensis* does not overtop the nutlets, whereas the stigma of *L. omphaloides* exceeds the fruit (Table 1). The relative length of the stigma vs. the nutlets represents a rather conservative character within *Lappula*. For example, taxa of Clade VIIb (*L. lasiocarpa*, *L. macrantha*, *L. semiglabra*, and *L. pavlovii*) have stigmas that do not overtop the fruit. Furthermore, individuals of *L. omphaloides* bear an appressed-sericeous-gray indumentum; this hair type is uncommon in *Lappula* and has hitherto been recorded only in *L. sericata* Popov (Clade VI) and *L. transalaica* (B. Fedtsch. ex Popov) Nabiev (Clade VIIa). In contrast, the remaining taxa of the clade containing *L. betpakdalensis* possess spreading hairs. Consequently, the morphological divergence between *L. betpakdalensis* and *L. omphaloides* far exceeds the expected range of intraspecific variation. Based on the morphological evidence from *L. omphaloides*, the taxon may belong to Clade VI or Clade VIIa. As material of *L. omphaloides* was not sampled in the present study, its phylogenetic position remains to be clarified by future molecular phylogenetic analyses.

Wang (1984) described *Lepechiniella lasiocarpa* based primarily on its saccate, densely pubescent nutlets. In their taxonomic revision of Chinese *Lappula*, Zhu et al. (1995) transferred *Lepechiniella lasiocarpa* to *Lappula*. Subsequent molecular phylogenetic studies confirmed this taxonomic placement, demonstrating that *L. lasiocarpa* belongs within *Lappula* (Liu et al. 2025a). However, Zhu et al. (1995) also treated *L. betpakdalensis* as a synonym of *L. lasiocarpa*. Morphological observations do not support this synonymization. Although both *L. betpakdalensis* and *L. lasiocarpa* possess distinctly winged nutlets, their wing morphology differs markedly: the wings of *L. betpakdalensis* are dentate, whereas those of *L. lasiocarpa* are entire (Fig. 2). Popov (1953a) regarded wing morphology as a key character for sectional delimitation within *Lepechiniella*, and this difference clearly exceeds the range of intraspecific variation. Furthermore, the two species also differ markedly in corolla size. The corolla of *L. betpakdalensis* is 3–4 mm in diameter, whereas that of *L. lasiocarpa* measures 5–6 mm. Together, these consistent morphological differences support the recognition of *L. betpakdalensis* and *L. lasiocarpa* as distinct species.

Phylogenomic analyses further support this conclusion. Although both the chloroplast and Angiosperms353 datasets place *L. betpakdalensis* and *L. lasiocarpa* within the same clade, the chloroplast phylogeny provides insufficient resolution to resolve relationships within Clade Fa, with bootstrap support values for most internal nodes below 50% (Fig. 1, Suppl. material 2: fig. S3–S5). Complete plastome sequences have been widely applied in Boraginaceae phylogenomics, including studies of Lithospermeae, *Arnebia* Forssk., and *Trigonotis* Steven (Park et al. 2020; Sun et al. 2022; Xu et al. 2023; Noroozi et al. 2024), where they generally recovered well-supported relationships. By contrast, plastome data provide relatively poor resolution of relationships among closely related *Lappula* species. This likely reflects the recent diversification of the genus, with most extant species diverging within approximately the last 3 million years (Sherafati et al. 2017), together with the relatively slow rate of plastome evolution (Wolfe et al. 1987). Consequently, plastome sequences may not have accumulated sufficient phylogenetically informative variation to resolve relationships among recently diverged species.

In addition, Liu et al. (2025a) demonstrated that incomplete lineage sorting, hybridization, and polyploidization have all contributed to the evolutionary history of *Lappula*. These processes can further reduce the congruence between plastid and species phylogenies. By contrast, nuclear loci from the Angiosperms353 probe set provide higher phylogenetic resolution and have been shown to resolve taxonomic relationships across Boraginaceae (Vasile et al. 2025; Meudt et al. 2025). In the present study, the phylogeny inferred from the Angiosperms353 nuclear loci clearly resolves *L. betpakdalensis* and *L. lasiocarpa* as two independent evolutionary lineages (Fig. 1). Accordingly, the nuclear phylogenomic evidence supports the recognition of these taxa as distinct species and does not support their previous synonymization.

Taken together, evidence from chloroplast and nuclear phylogenomics and detailed morphological comparisons demonstrates that *L. betpakdalensis* and *L. lasiocarpa* represent distinct species. Accordingly, the synonymization proposed by Zhu et al. (1995) is rejected, and *L. betpakdalensis* is reinstated as a distinct species.

### Taxonomic treatment

#### Lappula betpakdalensis

Taxon classificationPlantaeBoraginalesBoraginaceae

Nabiev, Opred. Rast. Sred. Azii 8: 138, 1986.

F22728B3-6F81-5ADD-81D5-1798121FF673

≡ Lepechiniella balchaschensis Popov, Spisok Rast. Gerb. Fl. S.S.S.R. 12: 50, 1953.
≡ Lepechiniella omphaloides var. *balchaschensis* Popov, Fl. URSS 19: 298, 1953.

##### Type.

Kazakhstan, clayey steppe on the northern shore of Lake Balkhash, 1884, *AM Nikolsky s.n*. (LE, Lectotype designated by Ovczinnikova, 2009).

##### Description.

Annual herbs. Stems erect, 10–30 cm tall, simple or branched from the base, spreading-pubescent. Basal leaves few, usually withering early, narrowly obovate, 1–2 cm long. Stem leaves sessile or the lower ones attenuate into a slender petiole, spatulate-linear to oblong-linear, flat, obtuse to acute, white-hispid on both adaxial and abaxial surfaces. Inflorescences elongating to 10 cm in fruit; bracts leaf-like at the base, gradually reduced upwards. Pedicels short, ca. 1 mm or less, erect, slightly stout, rarely arcuate. Calyx lobes narrowly ovate, obtuse, with subappressed hairs. Corolla blue; tube shorter than to nearly equalling the calyx; limb nearly flat, 3–4 mm in diameter, 5-lobed; lobes oblong to obovate. Style short, included between the nutlets. Nutlets heteromorphic. In broadly winged nutlets, the dorsal wing is saccate, and the wing margin is distinctly dentate, with glochids at the apex of each tooth. In narrowly winged nutlets, the wings are inconspicuous, and the margins bear glochids. Nutlets 2.2–2.7 mm long; disc narrowly lanceolate, smooth or tuberculate.

##### Phenology.

Flowering and fruiting May to June.

##### Distribution and habitat.

*L. betpakdalensis* is currently known only from the Lake Balkhash region of Kazakhstan.

##### Additional specimens examined.

• **Kazakhstan**, Karaganda Region, 24 May 1951, *NV Pavlov 327* (MW0876114!); Zhambyl Region, 14 May 2014, *AN Kupriyanov & IA Khrustalyova s.n*. (NSK0000034!); Zhambyl Region, 14 May 2014, A.L. Ebel s.n. (NSK0000037!); Bertys Bay, 15 June 1934, *MG Popov s.n*. (LE 01252312!, MW 0594322!, NSK0000085!). • **China**, Xinjiang, Hoboksar county, 21 May 2020, *DH Liu BNU2020XJ131*.

## Supplementary Material

XML Treatment for Lappula betpakdalensis

## Additional information

### Artificial Intelligence (AI) use

The author accept full responsibility for the content of the manuscript, including the disclosure of any use of AI.

Regarding the use of AI in the preparation of this manuscript, the authors declare the following: DeepSeek

Used for: Language, style and writing

### Author ORCIDs

D.-H. Liu https://orcid.org/0000-0002-0195-1436

### Data availability

All of the data that support the findings of this study are available in the main text or Supplementary Information.

## Supplementary materials

Supplementary material 1 Additional tables Data type xlsx https://binary.pensoft.net/file/1768422This dataset is made available under the Open Database License (http://opendatacommons.org/licenses/odbl/1.0/). The Open Database License (ODbL) is a license agreement intended to allow users to freely share, modify, and use this Dataset while maintaining this same freedom for others, provided that the original source and author(s) are credited. Dan-Hui Liu

Supplementary material 2 Additional figures Data type docx https://binary.pensoft.net/file/1768423This dataset is made available under the Open Database License (http://opendatacommons.org/licenses/odbl/1.0/). The Open Database License (ODbL) is a license agreement intended to allow users to freely share, modify, and use this Dataset while maintaining this same freedom for others, provided that the original source and author(s) are credited. Dan-Hui Liu
