# Supplementary material for: Reinstatement of Lappula betpakdalensis (Boraginaceae) based on phylogenomic and morphological evidence
Source: PhytoKeys. 2026 Sep 9;279:35–49. doi: 10.3897/phytokeys.279.207568 (PMC13584180; doi:10.3897/phytokeys.279.207568)
Supplement: Supplementary file 1 [file phytokeys-279-035_article-207568__-s002.docx]

**Supplementary Data Figures**

Supplementary Data Fig. S1. Heat map depicting recovery efficiency for Angiosperms353 genes enriched in Lappula as recovered by HybPiper. Each column represents a gene, and each row represents an individual sample. The shading in each cell corresponds to the proportion of sequence recovered, calculated as the length of the recovered sequence divided by the length of the reference gene (maximum value = 1.0).

Supplementary Data Fig. S2. Phylogenetic trees of *Lappula* inferred using concatenation methods in IQ‑TREE2 based on 274 Angiosperms353 genes. Numbers below the nodes represent bootstrap support (BS) values. The tree in the lower left corner includes branch lengths.

Supplementary Data Fig. S3. Phylogenetic trees of *Lappula* inferred in IQ‑TREE2 based on chloroplast genome. Numbers below the nodes represent bootstrap support (BS) values. The tree in the lower left corner includes branch lengths.

Supplementary Data Fig. S4. Phylogenetic trees of *Lappula* inferred in MrBayes based on chloroplast genome. Numbers below the nodes represent posterior probability (PP) values. The tree in the lower left corner includes branch lengths.

Supplementary Data Fig. S5. Phylogenetic trees of *Lappula* inferred in MrBayes based on 78 coding regions (CDS). Numbers below the nodes represent posterior probability (PP) values. The tree in the lower left corner includes branch lengths.

Supplementary Data Fig. S1

Supplementary Data Fig. S2

Supplementary Data Fig. S3

Supplementary Data Fig. S4

Supplementary Data Fig. S5
